# Editorial: The brain barriers in diseases of the nervous system

**DOI:** 10.3389/fncel.2023.1215651

**Published:** 2023-05-17

**Authors:** Marek Joukal, Lucy Vulchanova, Petr Dubový

**Affiliations:** ^1^Department of Anatomy, Faculty of Medicine, Masaryk University, Brno, Czechia; ^2^Department of Neuroscience, University of Minnesota, Minneapolis, MN, United States

**Keywords:** blood-brain barrier, blood-cerebrospinal fluid barrier, choroid plexus, drug delivery, meningeal blood-cerebrospinal fluid barrier, stroke

The central nervous system is protected against harmful substances from the blood by three major barriers: the blood–brain barrier, the choroid plexus blood–cerebrospinal fluid barrier, and the arachnoid blood–cerebrospinal fluid barrier. Under disease conditions (neurodegenerative, traumatic, oncologic, autoimmune), inflammatory responses within the brain barriers change their permeability. Therefore, the aim of this Research Topic was to provide new insights into (1) inflammatory responses within the brain barriers, (2) barrier changes associated with brain disorders, and (3) barrier modulation for drug delivery and treatment. This Research Topic consists of six articles, two reviews, and four original articles, written by authors with different expertise. All articles were focused on changes in the blood–brain barrier.

Alterations of the blood–brain barrier are associated with brain mass lesions such as glioblastoma, brain metastasis, and brain abscesses, which result in the formation of vasogenic brain edema. Cellular and molecular substrates of barrier disruption and edema formation have been extensively described in the review by Solar et al..

Disruption of the blood–brain barrier is also a significant component of the pathological processes that accompany ischemic stroke. Sphingosine-1phosphate (S1P) receptor 3 (S1PR3), as a member of the downstream G protein-coupled receptor family of S1P, was increased during cerebral ischemia/reperfusion (I/R) in mice (Fan et al.). After administration of the S1PR3-specific inhibitor CAY10444, the authors found decreased levels of zonula occludens 1 (ZO1) and occludin proteins as well as the amelioration of brain edema and neurological deficits.

It has been found that increased endothelial caveolae-mediated transcytosis, preceding blood–brain barrier disruption, is independent of TJs disintegration. Zhou et al. found that storax, a natural resin isolated from the wounded bark of Liquidambar orientalis, inhibited caveolae-mediated transcytosis in a rat focal stroke model and thus attenuated blood–brain barrier damage after stroke.

On the other hand, there is a necessity to find potential drugs that will increase the permeability of the blood–brain barrier. This effect is strongly needed for delivering therapeutics across the blood–brain barrier. It is well-known that bradykinin, an endogenous vasoactive peptide, can reversibly increase the permeability of the blood–brain and blood–tumor barrier (Sanovich et al., 1995; Borlongan and Emerich, 2003). However, bradykinin has limited value because of its short half-life and undesirable biological activity elicited by its active metabolites. Rodríguez-Massó et al. evaluated the effects of a stable bradykinin analog NG291 on blood–brain barrier permeability. They demonstrated that NG291-mediated blood–brain barrier disruption was localized, dose dependent, and reversible by measurement of Evans blue extravasation.

The pathophysiology of multiple sclerosis includes infiltration of the CNS by T helper lymphocytes, particularly Th1 and Th 17, which contribute to demyelination and neurodegeneration. The molecular mechanisms of the interactions between Th cells and CNS barriers were discussed in the review by Angelini et al., who were focused on the emerging roles of the dura mater and the arachnoid as neuroimmune interfaces contributing to the development of CNS inflammatory diseases. They proposed the restoration of the morphological integrity and functionality of CNS barriers as a promising therapeutic target to accelerate CNS recovery during multiple sclerosis.

Situ et al. investigated transcriptional changes in brain microvessels in a transgenic mouse model of cerebral amyloid angiopathy. They demonstrated that early inflammation manifested mainly by microglia/macrophage activation and the mediators of B lymphocytes' activities are the basic processes of blood–brain barrier hyperpermeability and cerebral microbleeding during the onset of cerebral amyloid angiopathy.

## Author contributions

All authors listed have made a substantial, direct, and intellectual contribution to the work and approved it for publication.
